# Ventromedial Prefrontal Cortex Is Critical for Helping Others Who Are Suffering

**DOI:** 10.3389/fneur.2018.00288

**Published:** 2018-05-25

**Authors:** Janelle N. Beadle, Sergio Paradiso, Daniel Tranel

**Affiliations:** ^1^Department of Gerontology, University of Nebraska at Omaha, Omaha, NE, United States; ^2^Private Practice in Psychiatry and Psychotherapy, Catania, Italy; ^3^Department of Neurology, Roy J. & Lucille A. Carver College of Medicine, University of Iowa, Iowa City, IA, United States; ^4^Department of Psychological and Brain Sciences, University of Iowa, Iowa City, IA, United States

**Keywords:** empathy, ventromedial prefrontal cortex, financial decision making, prosocial behavior, lesion study

## Abstract

Neurological patients with damage to the ventromedial prefrontal cortex (vmPFC) are reported to display reduced empathy toward others in their daily lives in clinical case studies. However, the empathic behavior of patients with damage to the vmPFC has not been measured experimentally in response to an empathy-eliciting event. This is important because characterizing the degree to which patients with damage to the vmPFC have lower empathic behavior will allow for the development of targeted interventions to improve patients’ social skills and in turn will help family members to better understand their impairments so they can provide appropriate supports. For the first time, we induced empathy using an ecologically-valid empathy induction in neurological patients with damage to the vmPFC and measured their empathic emotional responses and behavior in real time. Eight neurological patients with focal damage to the vmPFC were compared to demographically-matched brain-damaged and healthy comparison participants. Patients with damage to the vmPFC gave less money in the empathy condition to a person who was suffering (a confederate) than comparison participants. This provides the first direct experimental evidence that the vmPFC is critical for empathic behavior toward individuals who are suffering.

## Introduction

Daily we encounter people who are suffering—strangers living on the street; friends suffering from cancer who can’t pay their hospital bills; family members who have lost their homes to a fire. Traditional economic theories purport that we are rational actors who behave in ways that maximize our monetary gain, and therefore would be unlikely to donate to others in need (1). Yet, when people are asked to make financial decisions in daily life, researchers find that emotion (e.g., anger), not just rational thought, impacts our financial decisions toward others (2). A striking example of this can be seen in laboratory settings when people play economic decision making games, such as the Ultimatum Game (UG). When participants receive an offer that is perceived to be unfair, it is thought to elicit anger which in turn leads them to reject that offer, despite the negative financial impact of this choice (3).

From a neuroscience perspective, our financial decisions are thought to be guided by interacting brain systems involving cognition, emotion, and decision making (2, 4, 5). In fact, patients who have brain damage to a region implicated in decision making, the ventromedial prefrontal cortex (vmPFC), have difficulty making advantageous financial decisions (6, 7). In other words, their decisions result in financial outcomes that are poorer than that of healthy adults.

Clinical case studies demonstrate that patients with damage to the vmPFC have a reduced capacity to make decisions, ranging from minor decisions about choosing a restaurant, to major decisions about monetary investments (8–10). Furthermore, laboratory-based research studies show that patients with damage to the vmPFC have difficulty on multiple tasks measuring financial decision making (6, 11–14, 64). For instance, they have difficulty learning which decks are financially advantageous in the Iowa Gambling Task, and consequently achieve less overall financial gain than healthy comparison participants (11–13). In the UG, patients with damage to the vmPFC reject unfair offers at a higher rate than healthy comparison participants (6). This results in the patients obtaining less money overall than healthy adults. Based on these studies, researchers have hypothesized that patients’ decision making difficulties may derive from a reduced ability to utilize emotional information to guide decision making in an advantageous manner, as described by the somatic marker hypothesis (15–18).

Despite extensive research on financial decision making behavior in patients with damage to the vmPFC, we do not know how they behave in financial contexts where they witness another person who is suffering. This is an important question because many of our financial decisions occur in a social context. For example, a family member may need extra financial support if they develop a chronic illness, such as dementia. A long tradition of psychology and neuroscience research has characterized the behavior of healthy adults when they witness another person’s suffering (19–21). Research has shown that an antecedent to motivate someone to help another person is a perception or awareness that the person is in need of help (22). For instance, while there are situations that may evoke empathic joy toward others, such as when a best friend gets offered their dream job, this type of situation is not likely to elicit help because the person is not in need. Furthermore, extensive research has shown that feelings of empathy also motivate people to help others when they perceive them to be in need (20, 23–26).

Empathy is thought to be made up of two components: (1) cognitive—one’s ability to understand others’ thoughts and emotions, and (2) emotional—one’s ability to feel compassion and sympathy for the person in need or feel similarly to them (27). Individuals who experience high levels of empathy tend to show greater helping behaviors toward others in need than those experiencing low levels of empathy (20). Based on this body of research, the empathy-altruism hypothesis was developed which purports that empathic emotion is one mechanism for helping behavior toward others in need (23, 26).

Functional neuroimaging studies point to a broad network of brain regions involved in empathy, such as the vmPFC, amygdala, anterior cingulate, and anterior insula (28–31). Although functional neuroimaging studies provide important information about brain networks involved in empathy, lesion studies are able to determine which regions are critical for empathy to occur. There is a growing body of patient studies examining the degree to which lesions to regions including the anterior cingulate, insula, and amygdala affect empathy [(32–34); for review see Ref. (35)]. However, because only a small number of studies have investigated these regions using varying methodologies, currently there is no conclusive evidence that these regions are critical for empathy. In comparison, there is a long history of clinical and experimental research implicating the importance of the vmPFC for empathy (36–38). Therefore, due to the current state of the literature, we chose to focus on the vmPFC, because there is more substantial and consistent evidence that it is important for empathy.

Clinical case studies have shown that patients with damage to the vmPFC behave in ways that suggest they have reduced empathy toward others (39–41). However, these findings have not yet been demonstrated in a controlled, experimental context where participants with damage to the vmPFC show lower empathic behavior than healthy adults in response to an empathy-eliciting context. Furthermore, it has not yet been experimentally tested whether participants with damage to the vmPFC have reduced awareness of empathic information, reduced empathic emotion, or reductions in both domains in comparison to healthy adults. For empathic behavior towards others to occur, it is often motivated by both an awareness that the other person is in need and the experience of empathic emotion (22). If one or both these aspects are missing, the individual may exhibit lower empathic behavior. Therefore, assessing both the patients’ awareness of empathic information and their empathic emotion may aid in understanding potential motivations for their empathic behavior.

The information generated in the present study is crucial in designing effective interventions to improve social functioning in patients with damage to the vmPFC, because it will help clinicians to target the cognitive or emotional domains that are reduced in patients with damage to the vmPFC. If only their empathic behavior is lower than healthy adults, this can be targeted with behaviorally focused social skills training. If they are lower on their awareness of perceiving empathic information from empathy-eliciting contexts, they could receive training on how to determine when a situation is likely to evoke empathy in others. If they are lower on feelings of empathy, they could receive training on techniques to increase one’s empathy, such as imagining what the other person may be feeling. Furthermore, this is also important information for the patients’ family and caregivers because it will help them to better understand what social skills might be most difficult for the patients, so they can provide appropriate support. Therefore, the present study addresses a gap in the knowledge by experimentally investigating empathic behavior, empathic feelings, and awareness of empathic information in response to an empathy-eliciting context.

For the first time, the current study directly examines how patients with damage to the vmPFC behave in a financial context when exposed to someone who is suffering. The study uses a novel, ecologically valid empathy induction designed to represent a real-world scenario that would be likely to induce empathy. Furthermore, converging methods were used to assess empathy and financial decision making towards a man who is suffering. Specifically, these methods included (1) behavior—measure of financial decision making toward a suffering individual, (2) emotional response—real time patient self-reports of empathic emotion toward the suffering individual, (3) trait empathy—self-report assessing general tendency toward empathy in daily life completed by the patient (and patients’ family member), and (4) theory of mind—ability to accurately understand and assess others’ feelings and intentions.

The target group included eight neurological patients with focal damage to the vmPFC who were compared to a brain-damaged comparison (BDC) group and a healthy, normal comparison (NC) group. To reduce demand characteristics, participants were told that they would be playing an economic decision making game. During the course of the study, there was a neutral condition where the participant would overhear their opponent through the intercom talking about unemotional events from their day (e.g., playing a card game and reading the newspaper). The key target empathy induction condition involved the participant overhearing through the intercom system a second opponent discussing the anniversary of their son’s death and their grieving process. Empathic behavior was measured implicitly by how much money they gave to each opponent on the economic game (i.e., empathy versus neutral condition). To measure in the moment self-report ratings of empathy in response to the empathy induction, participants completed a mood questionnaire before and after each induction condition. This questionnaire measured empathy, in addition to other relevant emotions (e.g., sadness, hostility, joviality, and personal distress). At the end of the study, participants also completed a theory of mind task where they were asked to assess the intentions and feelings of others through written scenarios. Finally, participants completed a questionnaire measuring empathy as a general tendency across the lifespan which was also completed by their family members, as a means of corroboration.

It was hypothesized that patients with damage to the vmPFC will show significantly lower empathic behavior in response to an empathic induction in which they witness another person’s suffering than comparison groups. Furthermore, it was hypothesized that patients with damage to the vmPFC will show less empathic emotion than comparison participants in response to an empathy induction.

## Materials and Methods

### Participants

**Figure 1:**
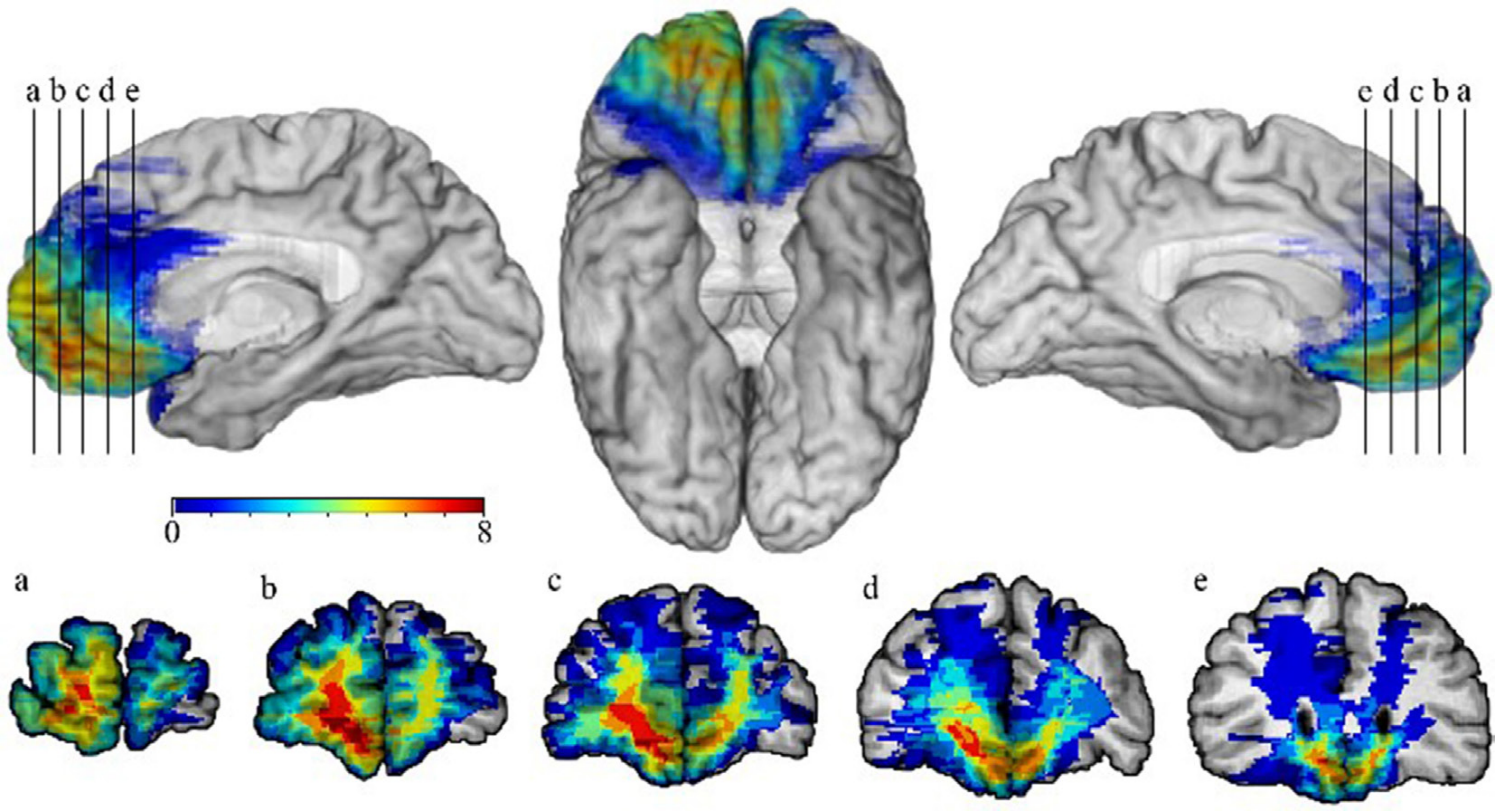
Lesion overlap map of patients with damage to the ventromedial prefrontal cortex (vmPFC). The lesion overlap map of eight patients with damage to the vmPFC is shown. Images are presented using radiological convention. Warmer colors indicate greater numbers of patients whose lesions overlap in a particular region, whereas cooler colors indicate fewer lesion overlaps. Overlap was greatest bilaterally in the ventromedial prefrontal region, with the cortex and white matter in the right vmPFC being involved in all eight patients.

Target participants included eight patients with focal damage to the vmPFC (see Figure 1). These patients were compared to NC (*N* = 8) and BDC (*N* = 8) groups. Comparison participants were matched to the target patients on age, education, gender, and full scale intelligence. All groups included five females and three males.

Kruskal–Wallis tests were used to compare age and education across the three groups. To compare chronicity between the BDC and vmPFC groups, a Mann–Whitney *U* test was used, and a Chi-square test was used to compare the two groups on type of etiology. In the present study, there were 19 statistical tests performed that were not testing *a priori* hypotheses. Therefore, we applied a false discovery rate correction for these tests (false discovery rate level: 0.05). There were no significant differences between groups on any of the demographic variables after the false discovery rate correction was applied [age: *X*(2) = 2.79, *p* = 0.25, Benjamini–Hochberg *p*-value = 0.59; education: *X*(2) = 4.94, *p* = 0.08; Benjamini–Hochberg *p*-value = 0.51; chronicity: *z*(14) = 2.53, *p* = 0.01, Benjamini–Hochberg *p*-value = 0.19; etiology: *X*(1) = 0, *p* = 1.00; Benjamini–Hochberg *p*-value = 1.00]. The BDC group included individuals with lesions outside of regions that have been previously implicated as being involved in empathy (Tables 1 and 2). In the BDC group, the lesions also excluded regions that have been associated with numeracy and valuation. The patients in the vmPFC group did not have major impairments in intelligence or memory, and they did not have premorbid personality disorders (6).^1^ Mann–Whitney *U* tests were used to compare the vmPFC group to the BDC group on relevant neuropsychological variables (WAIS-III Full Scale Intelligence Quotient—FSIQ, WAIS-III Working Memory Index—WMI, Trail Making Test Part A and B—TMT). There were no significant differences between the groups on any of the neuropsychological variables after the false discovery rate correction was applied [FSIQ: *z*(14) = 0.95, *p* = 0.34; Benjamini–Hochberg *p*-value = 0.68; WMI: *z*(14) = 0.74, *p* = 0.46, Benjamini–Hochberg *p*-value = 0.79; TMT-A: *z*(14) = 0.21, *p* = 0.83, Benjamini–Hochberg *p*-value = 0.97; TMT-B: *z*(14) = 0.00, *p* = 1.00; Benjamini–Hochberg *p*-value = 1.00]. This study was carried out in accordance with the recommendations of the Declaration of Helsinki and the University of Iowa Institutional Review Board with written informed consent from all subjects. All subjects gave written informed consent in accordance with the Declaration of Helsinki. The protocol was approved by the University of Iowa Institutional Review Board.

**Table 1:** Demographic characteristics of participants.

Group	vmPFC ID	Age (range)	Education (years)	Chronicity (years)	Etiology
	0770	66–70	16	24	Meningioma resection
	1983	46–50	13	14	Hemorrhagic stroke
	2352	60–65	14	11	Hemorrhagic stroke (SAH)
	2391	60–65	13	10	Meningioma resection
	2577	70–75	12	11	Hemorrhagic stroke (SAH)
	0318	66–70	14	34	Meningioma resection
	2025	56–60	16	14	Hemorrhagic stroke
	3001	60–65	14	7	Meningioma resection

vmPFC (*N* = 8)	M (SD)	62.4 (7.9)	14.0 (1.4)	15.6 (9.0)	4 Resection/4 stroke
	Median (range)	64.0 (46–70)	14.0 (12–16)	12.5 (7–34)	

BDC (*N* = 8)	M (SD)	58.0 (12.2)	13.6 (2.3)	7.0 (4.0)	4 Resection/4 stroke
	Median (range)	58.5 (44–75)	13.0 (11–18)	6.5 (3–16)	

NC (*N* = 8)	M (SD)	67.3 (7.5)	16.6 (3.0)	NA	NA
	Median (range)	67.5 (57–79)	17.0 (12–20)		

*Patients with damage to the ventromedial prefrontal cortex were case-matched on age, gender, education, and WAIS-III Full Scale Intelligence Quotient (FSIQ) to individuals from the two comparison groups. Chronicity, years between lesion onset and experimental testing session*.

*SAH, subarachnoid hemorrhage; vmPFC, patient with damage to the ventromedial prefrontal cortex; BDC, brain damaged comparison participant; NC, normal comparison participant; M, mean; SD, standard deviation; NA, not applicable*.

Kruskal–Wallis tests were used to compare age and education across the three groups. Mann–Whitney *U* test was used to compare chronicity between the BDC and vmPFC groups, and a Chi-square test was used to compare the type of etiology. There were no significant differences between groups on any of the demographic variables. Nineteen statistical tests were performed that were not testing *a priori* hypotheses. Consequently, we applied a false discovery rate correction for these 19 tests (false discovery rate level: 0.05). (To preserve the confidentiality of the patients who participated in the study, age is presented as a range.)

**Table 2:** Neuropsychological characteristics of patients.

Group	vmPFC ID	FSIQ	WMI	TMT A	TMT B
	0770	108	113	53	135
	1983	108	99	25	42
	2352	106	111	28	41
	2391	109	104	22	43
	2577	84	80	44	148
	0318	143	119	24	61
	2025	115	111	17	37
	3001	109	117	41	70

vmPFC (*N* = 8)	M (SD)	110.3 (16.1)	106.8 (12.6)	31.8 (12.7)	72.1 (44.4)
	Median (range)	108.5 (84–143)	111.0 (80–119)	26.5 (17–53)	52.0 (37–148)

BDC (*N* = 8)	M (SD)	107.8 (10.0)	105.1 (18.6)	37.1 (22.1)	86.3 (69.0)
	Median (range)	107.0 (97–129)	99.5 (86–133)	31.5 (18–77)	64.5 (30–221)

*FSIQ, WAIS-III Full Scale Intelligence Quotient; WMI, WAIS-III Working Memory Index; TMT A, Trail Making Test Part A; TMT B, Trail Making Test; vmPFC, patient with damage to the ventromedial prefrontal cortex; BDC, brain damaged comparison participant; M, mean; SD, standard deviation*.

*There were no significant differences between the groups on any of the neuropsychological variables when Mann–Whitney *U* tests were conducted. Nineteen statistical tests were performed that were not testing *a priori* hypotheses. Consequently, we applied a false discovery rate correction for these 19 tests (false discovery rate level: 0.05). NC group did not complete the neuropsychological testing portion of the study*.

### Experimental Design

The present experiment involved a quasi-experimental, cross-sectional design. The independent variables included experimental condition (neutral, empathy) and participant group (vmPFC patients, brain damage comparison patients, and normal, healthy adult comparison participants). The study used a within-subjects design, and thus all participants received both the neutral and empathy experimental conditions.

A novel empathy induction was used to elicit empathy in an implicit fashion similar to how empathy is frequently evoked in daily life—specifically, hearing another person talk about their struggles, frustration, and profound sadness. Participants were led to believe that the purpose of the study was to play an economic game (the UG) against a series of two opponents through an intercom system, with the opponents located in a different testing room. In one condition, *empathy induction*, the participant overheard (through the intercom) their opponent discussing the recent death of their son with the Research Assistant. In another condition (*neutral*, no empathy induction), the participant overheard their opponent discussing neutral, mundane events with the Research Assistant (e.g., such as playing cards or eating breakfast). Each participant underwent both the neutral and empathy induction in the same testing session. Due to the small number of available patients with damage to the vmPFC, the order of the inductions was not counterbalanced. The two opponents were actually audio recordings of community theater actors rather than real participants. The community theater actors were both males in their middle 50’s (chosen for having similar voice quality, age, and gender) and the Research Assistant in the study was a female in her 20’s. The age of the actors was selected to be similar to the age of the patient population in this study. Each audio recording was 4.5 min, with an 8-minute interval. This induction has effectively elicited empathy in healthy adults (42). For additional information on methods and pilot induction results see Ref. (43, 44).

### Empathic Behavior

Empathic behavior was measured as the difference between the amount of money offered to the opponent in the UG following the empathy induction and the amount offered in the neutral condition. In the UG, the participant decided how much to offer the opponent out of $10 on each of 20 rounds. The offers were summed across the 20 rounds, separately for each condition (empathy and neutral).

### Momentary Empathy and Emotional Responses

Empathy and other relevant emotions were measured through self-report momentary, state ratings that took place before and after each of the two conditions (neutral and empathy). Specifically, participants completed a questionnaire that assessed the participants’ momentary (or state level) of empathy, personal distress, joviality, hostility, and sadness. Participants were asked to respond to the prompt, “Indicate to what extent you feel this way right now, that is, at the present moment,” by rating each item on a scale from 1 (very slight or not at all) to 5 (extreme). This rating scale and prompt were adapted from the Positive and Negative Affective Schedule (PANAS) questionnaire (45). Furthermore, the items assessing joviality, hostility, and sadness were also adapted from this questionnaire and included sadness (“sad”; “downhearted”), hostility (“hostile”; “angry”), and joviality (“happy”; “joyful”). The items assessing emotional empathy and personal distress were drawn from a state measure of emotional empathy (23). These items included (“sympathetic”; “compassionate”) and (“upset” and “distressed”). These questionnaires have been used in previous research studies to measure state empathy, personal distress, and basic emotions in healthy adults and patients with brain damage (42, 46).

### Patients Thoughts and Feelings About Empathy Induction

We examined written free responses from the participants about their thoughts and feelings involving the empathy induction. This questionnaire was completed at the end of the experiment after the participant had undergone both the neutral and empathy conditions, but prior to the debriefing session about the purpose of the study. In particular, participants responded to a question about their thoughts and feelings in response to the empathy induction in which they overheard their second opponent in the game talking about the anniversary of their son’s death. Specifically the prompt was, “Please describe your thoughts and feelings (in a few words or a sentence) while hearing your second opponent talk with the Research Assistant. Please list these thoughts and feelings next to the bullets below. If there is not enough room, please use the lines below to describe further.”

These free responses were coded by two raters (research assistants) who were blind to the group each participant was assigned to as well as the purpose of the study. Responses were coded as a “1” if the written text mentioned at least one of the following terms: “sorry for,” “sad,” “sympathy/sympathetic,” and/or “compassion/compassionate.” Responses were coded as a “0” if the participant did not reference any of these terms.

### Believability/Manipulation Check

Participants completed four questions after the experiment, measuring the degree to which they believed they were playing against real opponents. The rating scale in response to these questions ranged from 1 = did not believe to 5 = believed extremely. These questions included the following: (1) “Did you believe that the first conversation you heard was a conversation between a Research Assistant and another person participating in the study?” (2) “Did you believe that the second conversation you heard was a conversation between a Research Assistant and another person participating in the study?” (3) “Did you believe that the first game was played against another person participating in the study?” (4) “Did you believe that the second game was played against another person participating in the study?” The responses across these four questions were averaged for each participant.

### Trait Empathy Ratings

Participants completed a questionnaire designed to measure empathy as a trait, or a general tendency in one’s daily life (27). In addition, the participants’ family members also completed the same trait questionnaire about the participants, as a means of comparison. (Not all family members of the participants were available to complete the questionnaires. The final sample of family members included a total of 14, across the three groups.)

The Interpersonal Reactivity Index (IRI) (27) was used to assess trait empathy and is a well-validated, multidimensional measure of empathy that assesses both the emotional and cognitive aspects of empathy. Emotional empathy was measured using the Empathic Concern subscale and cognitive empathy was assessed through the Perspective Taking subscale. Each subscale ranges from 0 to 28 points, and higher scores indicate a greater tendency towards empathy in daily life. The IRI has adequate test/retest reliability (range: *r* = 0.61–0.81) and internal consistency (range Cronbach’s alpha: 0.68–0.79). An example item from the questionnaire is, “When I’m upset at someone, I usually try to ‘put myself in his shoes’ for awhile.”

### Social Faux Pas Task: Assessing Accuracy of Detecting Others’ Intentions

Theory of mind was measured with a standard task assessing one’s ability to detect social faux pas from written scenarios, called the Social Faux Pas Task (47). In this task, participants read written scenarios about two characters engaged in a situation where someone says or does something that is socially inappropriate, or in other words, commits a social faux pas. Then, the participant answers a multiple choice question to determine whether they can detect what social faux pas was committed. In this task, there are also control scenarios to assess basic reasoning skills. A separate accuracy score is calculated for the 12 control and 12 theory of mind conditions for each participant.

### Statistical Analysis

#### Hypothesis Testing

Our primary variable of interest was the empathic behavior variable. We performed the Shapiro–Wilk test to assess the normality of the distribution of this variable. We found evidence that the NC group was not normally distributed (NC: S-W = 0.81, *p* = 0.04; BDC: S-W = 0.94, *p* = 0.59; vmPFC: S-W = 0.98, *p* = 0.97), and thus we have used non-parametric tests throughout this paper. We tested the degree to which the vmPFC group had lower empathic behavior than the comparison groups using a Kruskal–Wallis test. Next, we assessed the degree to which the vmPFC group had lower state empathy ratings than the comparison groups using a Kruskal–Wallis test. Based on our *a priori* hypotheses, planned comparisons (Mann–Whitney *U* tests) were used to compare each group to the other two groups on these variables.

#### Sample Description

The mean and standard deviation of the variables believability, trait empathy, theory of mind, and state emotions other than empathy (e.g., sadness, personal distress, hostility, and joviality) are presented in Table 3. Because we did not have specific hypotheses about these variables, these results are descriptive in nature. The exploratory analyses included separate Kruskal–Wallis tests to compare the three participant groups on believability, theory of mind, trait empathy (patient and family ratings), and each of the state emotions (i.e., sadness, personal distress, hostility, and joviality). If the result was significant at *p* < 0.05, Mann–Whitney *U* tests were used to assess differences between the groups. For all tests, uncorrected *p*-values are listed. A total of 19 statistical tests were performed that were not testing *a priori* hypotheses. Consequently, we applied a false discovery rate correction for these 19 tests (false discovery rate level: 0.05). We also list the Benjamini–Hochberg *p*-value that resulted from this false discovery rate correction. All statistical tests were two-tailed and findings were considered to be significant at the *p* < 0.05 level. Nonparametric tests were used for all analyses. For our qualitative exploratory analysis of patients’ thoughts and feelings in response to the empathy induction, we present the proportion of participants’ responses from each group that were coded as a 1 and the participants’ written responses. Statistics were not conducted on the thoughts and feelings responses because the results were qualitative in content.

**Table 3:** Assessments of state emotion, empathy, and theory of mind.

	vmPFC		BDC		NC		*p*-value
	M (SD)	Median (range)	M (SD)	Median (range)	M (SD)	Median (range)	
**State emotion ratings**							
Empathy	0.9 (1.0)	0.8 (0–3)	2.0 (1.4)	2.0 (0–4)	1.8 (1.3)	2.3 (0–3)	0.21
Sadness	0.6 (0.4)	0.8 (0–1)	1.2 (1.0)	1.0 (0–2.5)	0.5 (0.6)	0.3 (0–1.5)	0.24
Personal distress	0.3 (0.3)	0.3 (0–0.5)	0.1 (0.5)	0 (−0.5–1)	0.2 (0.6)	0 (−0.5–1.5)	0.65
Hostility	−0.2 (0.4)	0 (−1–0)	0 (0)	0 (0–0)	0 (0)	0 (0–0)	0.12
Joviality	−0.3 (0.8)	0 (−1.5–0.5)	−0.6 (0.9)	−0.5 (−2.5–0.5)	−0.8 (0.9)	−0.5 (−2.5–0)	0.57

**Trait empathy ratings**							
*IRI-Perspective Taking (cognitive empathy)*							
Participants	18.5 (3.7)	18.0 (14–26)	19.8 (4.5)	20.0 (14–26)	17.1 (3.8)	17.0 (12–24)	0.52
Family	14.0 (6.8)	12.5 (8–27)	16.3 (3.9)	16.5 (12–20)	20.0 (2.6)	20.0 (17–23)	0.14
Difference score	−4.5 (8.0)	−3.0 (−16–7)	−1.8 (6.4)	−1.0 (−10–5)	1.5 (3.7)	0 (−1–7)	0.27
*IRI-Empathic Concern (emotional empathy)*							
Participants	22.1 (3.9)	23.0 (14–26)	20.6 (4.5)	20.0 (15–28)	23.5 (2.0)	23.0 (20–27)	0.36
Family	19.2 (5.5)	18.5 (13–26)	19.0 (2.9)	18.5 (16–23)	23.8 (4.0)	25.0 (18–27)	0.21
Difference score	−2.8 (4.6)	−1.8 (−11–3)	−1.8 (5.0)	0 (−9–2)	−0.5 (3.9)	0.5 (−6–3)	0.55

**Theory of mind task: Accuracy (%)**							
Theory of mind	82.3 (14.4)	87.5 (50–91.7)	62.5 (19.4)	62.5 (33.3–91.7)	83.3 (6.3)	83.3 (75–92)	0.05
Control	77.1 (13.2)	70.8 (66.7–100)	80.2 (12.5)	83.3 (66.7–100.0)	83.3 (8.9)	83.3 (75–100)	0.58

*Group labels include: vmPFC, ventromedial prefrontal cortex; BDC, group of patients with damage to areas of the brain not related to empathy; NC, healthy adult normal comparison group; M, mean; SD, standard deviation. State emotion rating change scores represent the effect of the empathy induction on each emotional state by subtracting out their emotional response to the neutral condition and their baseline response. IRI, Interpersonal Reactivity Index. Participants indicates participants’ self-reported score on the questionnaire. Family indicates family member ratings of the participant. Difference score indicates participant score was subtracted from family member score—negative scores indicate family member rated the participant lower than the participant rated themselves; positive scores indicate family member rated the participant higher than the participant rated themselves. Theory of mind task represents accuracy on the theory of mind condition, control indicates accuracy on the control condition. Kruskal–Wallis tests were used to compare the three groups on each measure. Because the state empathy rating examined a specific hypothesis, planned comparison tests were used, with no correction. Nineteen statistical tests were performed that were not testing *a priori* hypotheses. Consequently, we applied a false discovery rate correction for these 19 tests (false discovery rate level: 0.05)*.

## Results

### Hypothesis Testing

#### Primary Behavioral Analysis: Empathic Behavior Towards a Suffering Individual

**Figure 2:**
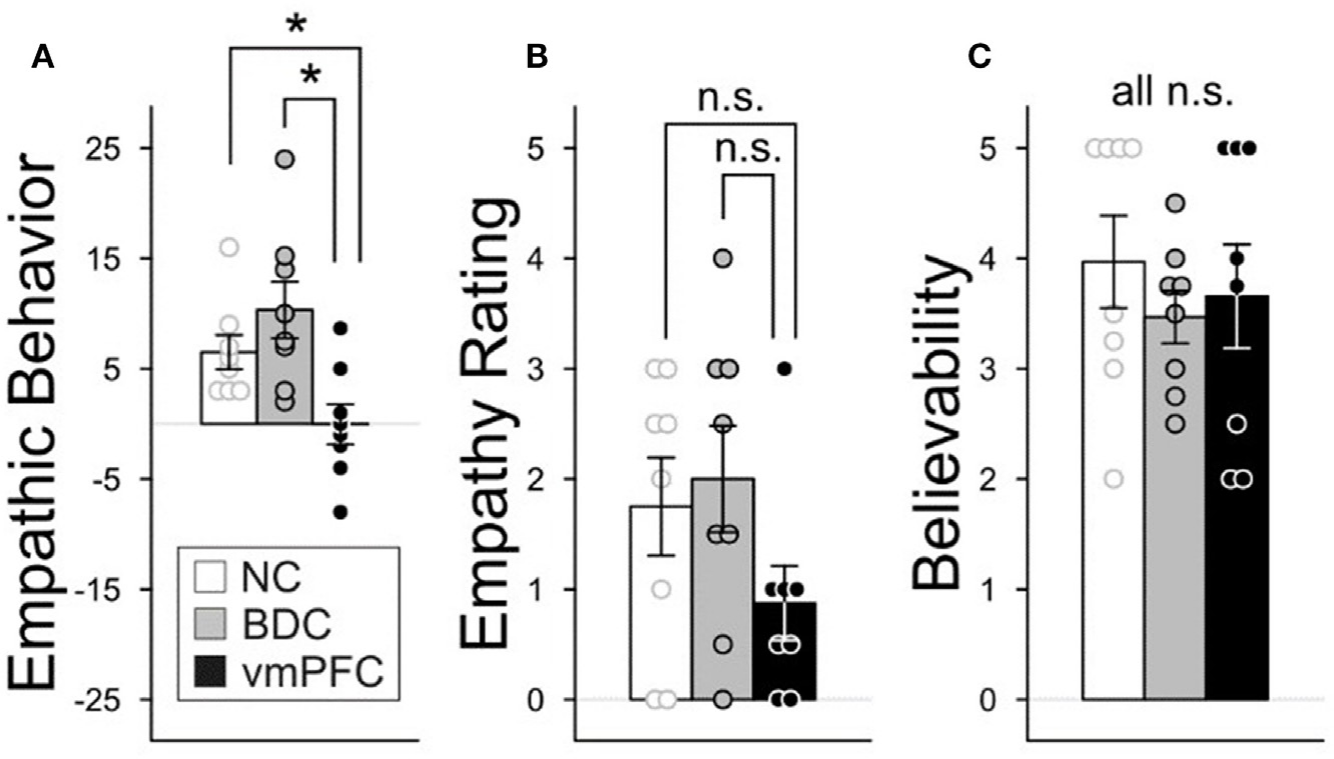
Group differences in empathic behavior, ratings, and believability. The three participant groups were compared on their empathic behavior, ratings, and the believability of the experiment. Graphs depict mean values and error bars are standard error of the mean. *p < 0.05. N.S., not significant. **(A)** Empathic behavior by group. Empathic behavior on the Ultimatum Game (UG) was depicted as a change score reflecting the difference in the amount of money given after experiencing an empathy or neutral condition (sum of offers: empathy – neutral condition). Positive numbers indicate that greater money was given in response to the empathy induction than the neutral condition. **(B)** Empathy ratings by group. An empathy rating change score was computed measuring empathic concern ratings before and after each induction condition: (After – Before Empathy Induction) – (After – Before Neutral Induction). Positive change scores indicate higher ratings on the empathy induction versus the neutral condition. **(C)** Believability. Participants completed four questions at the end of the experiment measuring the degree to which they believed they were playing against real opponents. Responses across the four questions were averaged. (Rating scale: 1–5; 1 = did not believe and 5 = believed extremely.) Nineteen statistical tests were performed that were not testing *a priori* hypotheses. Consequently, we applied a false discovery rate correction for these 19 tests (false discovery rate level: 0.05).

The primary analysis addressed the degree to which participants demonstrated empathy behaviorally by making larger offers in the UG in response to the empathy condition in comparison to the neutral condition. Patients with damage to the vmPFC did not make higher offers in the empathy condition than in the neutral condition, whereas both comparison groups made much higher offers [Figure 2A, group: *X*(2) = 9.56, *p* = 0.008; follow-up planned comparisons: vmPFC vs. BDC: *z*(14) = 2.73, *p* = 0.006; vmPFC vs. NC: *z*(14) = 2.37, *p* = 0.02; BDC vs. NC: *z*(14) = 1.06, *p* = 0.29]. The range of offers in each group included: vmPFC = −$8 to 8.67^2^; BDC = $2 to 24; NC = $3 to 16. In fact, of the eight vmPFC patients, four patients actually gave lower offers to the man who had lost his son, two had virtually zero change, and two had increases in response to the empathy condition. In sharp contrast, all 16 participants in the comparison groups gave higher offers in response to the empathy induction; in many instances, these were much higher (Figure 2A; Figure S1 in Supplementary Material for additional information).

#### Emotional Response: State Empathy

We compared the degree to which there were differences in state self-rated empathy in response to the experimental conditions across the three groups. Overall, group differences were not significant (Figure 2B; Table 3, *X*(2) = 3.11, *p* = 0.21). Follow-up planned comparisons revealed that the vmPFC group did not significantly differ from the BDC group [*z*(14) = 1.70, *p* = 0.09] or from the NC group [*z*(14) = 1.23, *p* = 0.22]. Also, the BDC and NC groups did not differ significantly [*z*(14) = 0.43, *p* = 0.67].

We tested the degree to which the state empathy of the three groups was statistically equivalent using the two one-sided tests (TOST) procedure (48), with an alpha level of 0.05 and an effect size value of Cohen’s *d* = 0.3 (indicating a small effect size). When comparing the vmPFC group to the BDC group, the equivalence test was non-significant [*t*(12.54) = 1.31, *p* = 0.89]. When comparing the vmPFC group to the NC group, the equivalence test also was non-significant [*t*(13.08) = 0.97, *p* = 0.83]. When comparing the BDC group to the NC group, the equivalence test was non-significant [*t*(13.90) = −0.22, *p* = 0.42]. Although there were no statistically significant differences between the groups on this measure, our study’s small sample size prevented us from establishing statistically significant equivalence between the groups.

### Exploratory Analyses

#### Patients Thoughts and Feelings About Empathy Induction

We sought to further understand the degree to which patients were aware that the content of the empathy induction was about an empathy-eliciting situation. To further assess this question, we examined written free responses from the participants in response to a questionnaire that occurred at the end of the experiment, but prior to the debriefing session about the purpose of the study. In particular, participants responded to a question about their thoughts and feelings in response to the empathy induction in which they overheard their second opponent in the game talking about the anniversary of their son’s death. Specifically, the prompt was, “Please describe your thoughts and feelings (in a few words or a sentence) while hearing your second opponent talk with the Research Assistant. Please list these thoughts and feelings next to the bullets below. If there is not enough room, please use the lines below to describe further.” We present the full written responses of the patients with damage to the vmPFC in Table 4. The full written responses of the BDC and NC groups are presented in Table S1 in Supplementary Material.

**Table 4:** Written free responses about empathy induction by patients with damage to the ventromedial prefrontal cortex (vmPFC).

770	“He seemed to be an ordinary person well-adjusted until he started talking about the death of son and it made me feel sorry until he said the death was not his fault and he did have ideas of how to overcome his loss and I feel he is in control and things will improve as time goes on.”

1983	“Again, why are they doing research? How old is this person? Did they have some kind of brain trauma? Do they wonder about me?”

2352	“Sad person since son’s death; could not connect with wife’s feelings now; desperately looking for help.”

2391	“Sympathy for losing a loved one; compassion for what he is experiencing. My brother died on [excluded for confidentiality]. I have experienced the death of a loved one, so I can relate to how he is feeling. He has a long way to go before his son’s death won’t hurt.”

2577	“Sadness with loss of loved one.”

318	“He is emotional, sad, articulate. He articulates and evaluates such strong emotion very well.”

2025	“I’ve never played bridge. How extremely sad that son died. I’d like to suggest he find a support group.”

3001	“He is not dealing well with the loss of his son. He is trying to get beyond the loss of his son. This loss is effecting him daily. I feel compassion for him and his wife.”

*Participants with damage to the vmPFC filled out a questionnaire after the experiment was completed about their thoughts and feelings in response to the audio recording designed to induce empathy. Specifically, participants responded to the prompt: “Please describe your thoughts and feelings (in a few words or a sentence) while hearing your second opponent talk with the Research Assistant. Please list these thoughts and feelings next to the bullets below. If there is not enough room, please use the lines below to describe further.” We list the written comments of each participant with damage to the vmPFC*.

Two raters who were blind to the group each participant was assigned to as well as the purpose of the study coded the written free responses of the participants. Responses were coded as a “1” if the written text mentioned at least one of the following terms: “sorry for,” “sad,” “sympathy/sympathetic,” and/or “compassion/compassionate.” Responses were coded as a “0” if the participant did not reference any of these terms. In the group of patients with damage to the vmPFC, seven out of the eight patients’ responses were coded as a “1” in response to the empathy condition. Similarly, in the BDC group, seven out of the eight patients’ responses were coded as a “1” and in the NC group, all eight participants were coded as a “1.” (There was perfect agreement among the raters in their coding of the written responses). Some examples of the free responses of the patients with damage to the vmPFC are, “Sad person since son’s death; could not connect with wife’s feelings now; desperately looking for help,” and, “Sympathy for losing a loved one; compassion for what he is experiencing.”

#### Manipulation Check

A manipulation check was used to determine the degree to which participants believed the experiment (i.e., whether participants believed that the opponents they overheard during the experiment through the intercom were actual participants). This manipulation check demonstrated that the groups did not significantly differ on the believability measure [*X*(2) = 0.87, *p* = 0.65; Benjamini–Hochberg *p*-value = 0.82]. For additional information about the believability results and questionnaire, see Figure 2C and Section “Materials and Methods.”

#### State Emotion

There were no significant group differences after a false discovery rate correction in any of the emotions measured in response to the experimental conditions which included sadness, personal distress, hostility, and joviality [sadness: *X*(2) = 2.87, *p* = 0.24; Benjamini–Hochberg *p*-value = 0.59; personal distress: *X*(2) = 0.87, *p* = 0.65, Benjamini–Hochberg *p*-value = 0.97; hostility: *X*(2) = 4.17, *p* = 0.12, Benjamini–Hochberg *p*-value = 0.53; joviality: *X*(2) = 1.12, *p* = 0.57, Benjamini–Hochberg *p*-value = 0.79; (Table 3)].

#### Trait Empathy and Accuracy of Assessing Others’ Intentions

Groups were compared on their self-reported trait empathy (cognitive—IRI Perspective Taking subscale; emotional—IRI Empathic Concern subscale), and their theory of mind performance (Table 3; see Materials and Methods). In addition, family members completed the trait questionnaire about the participants, as a means of comparison. This analysis revealed no significant differences between the groups in self-reported trait empathy by the participants after false discovery rate correction [IRI-EC: *X*(2) = 2.07, *p* = 0.36; Benjamini–Hochberg *p*-value = 0.68; IRI-PT: *X*(2) = 1.31, *p* = 0.52, Benjamini–Hochberg *p*-value = 0.79]. There were also no significant differences between the groups after false discovery rate correction in family members’ reports of participants’ trait empathy [Table 3; IRI-EC: *X*(2) = 3.16, *p* = 0.21, Benjamini–Hochberg *p*-value = 0.59; IRI-PT: *X*(2) = 3.89, *p* = 0.14, Benjamini–Hochberg *p*-value = 0.53].

Next, the groups were compared on their accuracy scores in the theory of mind task (Social Faux Pas Task) which measures one’s ability to detect the motivations and intentions of others through written scenarios. This task includes a theory of mind condition (i.e., accuracy of determining others’ intentions) and a control condition (i.e., accuracy of basic reasoning skills). When comparing the performance accuracy of the groups on the theory of mind condition, there was no significant effect of group after correction for false discover rate [Theory of mind condition: *X*(2) = 5.82, *p* = 0.05, Benjamini–Hochberg *p*-value = 0.48]. There were also no significant group differences in the control condition after correction for false discovery rate [Control condition: *X*(2) = 1.11, *p* = 0.58, Benjamini–Hochberg *p*-value = 0.79].

## Discussion

For the first time, we experimentally demonstrated that patients with damage to the vmPFC behaved with little empathy in a financial context towards a man who is suffering. This corroborates clinical case studies reporting that patients with damage to the vmPFC behave with reduced empathy toward family members (40, 41, 49). Furthermore, we also advance the literature by demonstrating that patients with damage to the vmPFC obtained more money than comparison groups in the UG when witnessing another person suffering. This is in contrast to previous studies which found that patients with damage to the vmPFC achieved poorer financial outcomes than comparison participants in decision making games, such as the Iowa Gambling Task (11, 12). The findings of the present study are consistent with research suggesting that the vmPFC plays an important role in using contextual information to guide decision making (50, 51).

Patients with damage to the vmPFC showed significantly less empathic behavior towards a person who was suffering. Specifically, the patients with damage to the vmPFC did not give more money to the man who was suffering than to the man in the control condition. In stark contrast, the comparison groups gave more money to the man who was suffering than to the man in the control condition. However, behaving with less empathic behavior than the comparison groups actually benefited the patients with damage to the vmPFC financially, as they received higher rather than lower financial payoffs than comparison participants.

Determining whether a financial decision is advantageous or not depends not only on the financial outcome, but also on the social consequences that may result. For instance, imagine the situation in which your mother cannot afford her chemotherapy treatments. You decide to help pay for her chemotherapy treatments, even though this decision could negatively impact your financial situation out of concern for her well-being which may in turn result in increased relationship quality. Therefore, healthy adults may choose to forego financial gain in order to achieve greater social rewards. On the other hand, imagine if an individual acted in a manner similar to the patients with vmPFC damage in the current study where they decided to pay very little for the chemotherapy treatments. Although this would result in better financial outcomes for the patient, it could severely and negatively impact their relationship with their mother. Therefore, advantageous financial decision making in social contexts is likely to require making decisions that are likely to facilitate social relationships, even if finances are negatively impacted.

The lower empathic behavior of patients with damage to the vmPFC in the present study may contribute to their difficulties making and maintaining relationships that are often highlighted in case reports (8, 9, 52). Anderson and colleagues studied two cases of patients with damage to the vmPFC and noticed that they both had few friends, mentioning that in the case of Patient B the, “lack of friends was conspicuous,” (52). Furthermore, there is other anecdotal evidence that patients with damage to the vmPFC have difficulty maintaining relationships, such as in the case of seminal patient EVR, who went through a divorce after 17 years of marriage (8), and in another case study of a patient who had already been divorced by 21 years of age (9). Because of the important role of empathic behavior in maintaining and nurturing relationships, it is likely that difficulty showing empathic behavior toward others could have a negative impact on one’s personal relationships.

The results in the present study also provide new information about how patients with damage to the vmPFC perceive and experience empathy-eliciting situations. We report that patients with damage to the vmPFC did not significantly differ from the comparison groups in their experience of “in the moment” empathy in response to an empathy induction involving exposure to another person’s suffering. However, it should be noted that our study’s small sample size prevented us from establishing statistically significant equivalence between the groups. Consequently, at this time, we cannot determine whether the patients with damage to the vmPFC have lower or equivalent levels of state empathy relative to the comparison groups. Our exploratory free response analyses suggest that the majority of patients with damage to the vmPFC are aware that they were exposed to an empathy-eliciting situation, as seven out of the eight patients reported that they felt “sorry for,” “sad,” “sympathy/sympathetic,” and/or “compassion/compassionate” in response to the empathy induction. However, we note that despite this reported awareness of the empathic content, this was not sufficient for the patients with damage to the vmPFC to behave in an empathic manner. Taken together, our findings suggest that future studies are needed to tease apart this important question as to whether patients with damage to the vmPFC have lower state empathy than healthy adults in response to empathy-eliciting events.

The patients with damage to the vmPFC in our sample did not significantly differ from the comparison groups in their ability to accurately detect the intentions of others in a separate theory of mind task. A previous study of patients with damage to the vmPFC focused on patients’ reports of trait empathy and found that there is evidence for lower reported cognitive empathy than healthy adults of comparable demographics (38). However, a key difference between the present study and study by Shamay-Tsoory and colleagues is that their sample of patients with damage to the vmPFC included a large proportion of closed head injury cases which could have had more diffuse brain damage. In contrast, the present sample does not include any closed head injury cases. Furthermore, in the present study, we did not find significant group differences in accuracy on the theory of mind task which would provide further support that their ability to discern others’ intentions is relatively intact. Previous studies of theory of mind in patients with damage to the vmPFC have found mixed evidence about whether they have difficulty detecting others’ intentions and motivations (53, 54).

It is relevant to discuss our findings in the context of important theories of vmPFC function [for review see Ref. (55)]. The somatic marker hypothesis proposes the role of the vmPFC as a secondary inducer, or a higher order emotional response that helps to guide decision making (17, 18). Roy and colleagues highlight an important role for the vmPFC in affective meaning (56). In particular, they suggest that the vmPFC plays a role in behavioral responses to higher order conceptual levels of emotion, rather than lower order simple emotional responses (56). The role for the vmPFC in insight and reflection has also been pointed out by Koenigs et al. (57) who suggested that this region plays an important function in reflecting on one’s emotional state and how it may affect others. Our results suggest that the vmPFC is important for behaving in an empathic manner in response to a financial context in which another person is suffering. We find preliminary evidence that despite seven out of eight patients reporting that they are aware of the empathic context, they did not show empathic behavior, suggesting that they have difficulty using this type of information to guide their empathic behavior.

In the financial domain, previous studies have established that the vmPFC is critical for advantageous financial decisions, whether in the context of the Iowa Gambling Task (15, 16), or the UG (57). In both cases, patients with vmPFC damage act differently than normal healthy adults, and fail to use emotional information in an advantageous way to guide financial behavior. In the present study, the patients with damage to the vmPFC appear to be aware of the empathy-eliciting context but behave with lower empathy than the comparison groups. However, as a result, they also have greater payoffs in the game. Consequently, it suggests that the vmPFC may be important for using contextual information in a socially advantageous manner, such as showing empathy toward others in need, or regulating one’s anger when someone rejects your offers in the UG. This interpretation is in line with Koenigs et al.’s (57) discussion of the important role for the vmPFC in self-reflection about one’s emotions and the consequences of their behavior. Although the present study focuses on the financial domain, the vmPFC may serve similar functions in non-financial contexts. This is seen in their failure to use emotional context in a socially advantageous manner in moral scenarios, as patients with vmPFC damage exhibit utilitarian type behavior (58) and socially inappropriate behaviors (59). In summary, the present study adds to the growing literature on the role of the vmPFC in social decision making in financial and non-financial contexts.

This study has limitations. The measure of empathic feelings in this study was self-report which can be influenced by concerns for social desirability. To attempt to address this issue, we also collected ratings from the family members about the patients’ empathy, as a form of corroboration. We found that the family members’ ratings did not significantly differ across the participant groups, which provides support for the accuracy of the patient ratings. In the present study, we cannot directly address the question of whether the empathy that the patients with damage to the vmPFC felt in response to the empathy induction was similar to or more extreme than the level of empathy they may experience in their daily lives. To answer this question, future studies may compare patient ratings of empathy in real time in their daily lives vs. laboratory-based empathy inductions. Because patients with focal damage to the vmPFC are rare, our sample size is smaller than that of studies focusing on healthy adults. However, the size of our sample is consistent with other studies on patients with damage to the vmPFC [e.g., *N* = 7, (6); *N* = 8, (58)]. Because the inductions were not counterbalanced in the present study, there is the possibility of an order effect. In a different study of healthy younger and older adults, we counterbalanced the order of a similar empathy induction and neutral induction (this one used a series of notes rather than audio recordings) and found no significant effects of order. This suggests that in a similar context, there was no significant effect of order (46). However, in future studies, it would be useful to counterbalance the order of the conditions in order to specifically address this limitation. Characterization of the patients’ emotional responses to empathy inductions through physiological (e.g., skin conductance, heart rate) measures would further add to our understanding of their momentary empathic experience in response to others’ suffering.

In summary, the current study is the first to experimentally demonstrate that the vmPFC is critical for empathic behavior in a financial context towards those who are suffering. We show preliminary evidence that awareness of an empathy-eliciting event, where someone is suffering, is not enough to elicit empathic behavior in patients with damage to the vmPFC. Rather, it suggests that these patients do not appear to use this information to guide their behavior in a way that helps the suffering person. On the other hand, by behaving in a manner seemingly not influenced by the empathic context, patients with damage to the vmPFC have better financial payoffs than the comparison groups.

These findings have broad implications for the treatment of other populations suffering from difficulty behaving with empathy toward others who are suffering. It helps us understand how groups affected by changes to the frontal lobe might respond in financial contexts where they witness another person’s suffering. Because decreased functioning of the frontal lobe is seen in many different populations ranging from healthy aging, to dementia, and brain injury, it has far reaching implications for financial decision making in social contexts for these groups (60). This is important for family members of patients with damage to the frontal lobe to be aware of because it may help them to have greater compassion for the patient.

In contrast to the behavior of the patients with damage to the vmPFC, if an individual puts too much weight on the emotional context of a situation, it could also have a negative impact on financial decisions and personal relationships. For instance, highly empathic caregivers or nurses may become too emotionally invested in their patients or loved ones which could lead to compassion fatigue and burnout (61–63). In future research studies, it would be useful to investigate the utility of interventions designed to help individuals strategize about making financial decisions that optimize both financial and social well-being. In conclusion, the present study characterizes the role of the vmPFC in an empathy-eliciting situation involving financial decision making towards an individual who is suffering.

## Ethics Statement

This study was carried out in accordance with the recommendations of the Declaration of Helsinki and the University of Iowa Institutional Review Board with written informed consent from all subjects. All subjects gave written informed consent in accordance with the Declaration of Helsinki. The protocol was approved by the University of Iowa Institutional Review Board.

## Author Contributions

JB was involved in the methodological design, data collection and analysis, theoretical framework, and writing the manuscript. DT and SP contributed to the methodological design, theoretical framework, and writing the manuscript.

## Conflict of Interest Statement

The authors declare that the research was conducted in the absence of any commercial or financial relationships that could be construed as a potential conflict of interest.

## Footnotes

^1^Some of the target participants previously participated in another study from our laboratory examining the 1-shot version of the UG in the role of the Responder, the person who decides whether to accept or reject the offer. These include patients 1983, 0770, 0318, 2577, 2352, and 2391. This previous study did not include an empathy induction. In the present study, we investigate patients’ responses to an empathy induction on the UG in the role of the Proposer, the person who makes the offer.

^2^A fraction is indicated here because one patient with damage to the vmPFC was missing one offer, and thus a mean substitution approach was used to approximate the missing value based on the patient’s other offers.
