# Supplementary material for: Ventromedial Prefrontal Cortex Is Critical for Helping Others Who Are Suffering
Source: Front Neurol. 2018 May 25;9:288. doi: 10.3389/fneur.2018.00288 (PMC5981225; doi:10.3389/fneur.2018.00288)
Supplement: Supplementary file 1 [file Image_1.PDF]

Supplementary Figure Legend

Supplementary Fig. 1. Effect of Empathy Induction on Financial Decision Making by Group. This graph descriptively depicts the amount of money given, on average, per condition (neutral or empathy) and per group (ventromedial prefrontal cortex=vmPFC, brain damage comparison participant=BDC, and normal comparison participant=NC). Error bars reflect standard error.

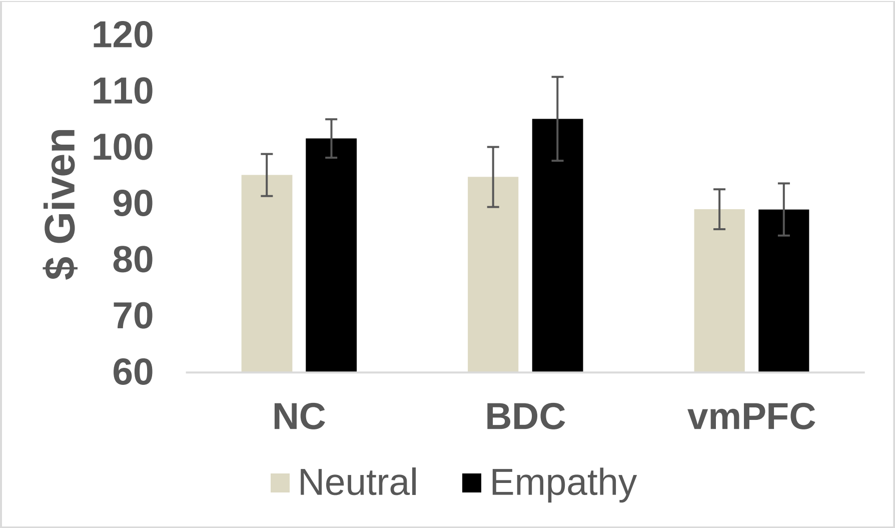

Supplementary Fig. 1. Effect of Empathy Induction on Financial Decision Making by Group
