# Supplementary material for: Ventromedial Prefrontal Cortex Is Critical for Helping Others Who Are Suffering
Source: Front Neurol. 2018 May 25;9:288. doi: 10.3389/fneur.2018.00288 (PMC5981225; doi:10.3389/fneur.2018.00288)
Supplement: Supplementary file 2 [file Table_1.PDF]

Supplementary Table 1.

**BDC Group**	
2843	"Very sad story!! The game was "distraction" for him. I wanted him to win."
1861	"I felt very sorry for him and understood his feelings. I lost a daughter 5 years ago and know just how he felt. Hope he and his wife understand son would want him and his wife to think about the joy his son gave them while growing up."
3353	"Felt empathy for him. Wanted to give a hug to him and his wife. Wanted to tell him to talk about son's death. Remember the positive things about his son. How his son brought him joy and peace. Talk to a 3rd person/or dear friend openly. Don't let sadness build up inside."
3105	"Compassion. Sorrow. Thoughtfulness. Warmth."
2994	"Friendly individual. Bridge player- a thinker! Sadness- sympathy. Compassionate."
3379	"I am very sorry for him and his family. I wish him the best in the game. I know what he is going through."
3177	"Sad. Nice. Heartbroken. Wanted to give him a hug. Other: opponent sounded sad when he talked about his son dying. I hate a when bad things happen to nice people."
2962	"Sounded like a confident player. Played the game to win. Would not play the game to lose."
**NC Group**	
179	"Sensed a lot of apprehension in speaker. Alarm mounted as to whether he should be doing the study. Great sympathy for his loss. Amazed that he should open up quite so much to a stranger."
1052	"Sad for him. Empathetic. Encouraging. Hopeful."
111	"Glad he has bridge to take away sad though. Sorry about the death of his son. Can[']t believe that he is able to share his feelings. Is this the same person as the first?"
1049	"Felt sad, for his situation, what a difficult day this must be for him. I wonder why he'd do this game today. I hope he is able to find a friend to talk to and get through the day."
106	"He sounded sad and confused. He needed someone to talk to. I felt sad and wanted to talk to him."
132	"Sad over anniversary of death of son. Real compassion. Willing to submit feelings to him."
216	"Opponent started off relaxed. Opponent was soft spoken. Opponent began to become somewhat distressed as he recalled the "death of this child." Other: opponent had similar positive traits to the first opponent but because of his manifested grief I played the game even a bit more compassionately than I did with opponent #1."
126	"Compassion. Empathy. Then, not sure if it was staged or not. Certainly changed my feelings. Other: still not sure whether it was real or acted to play on my emotions."

Note. Participants completed a questionnaire after the experiment was completed about their thoughts and feelings in response to the audio recording designed to induce empathy.

Specifically, participants responded to the prompt: “Please describe your thoughts and feelings (in a few words or a sentence) while hearing your second opponent talk with the Research Assistant. Please list these thoughts and feelings next to the bullets below. If there is not enough room, please use the lines below to describe further.” We list the written comments of each participant by group. Labels: BDC Group=indicates brain damage comparison participant group. NC Group=indicates normal comparison participant group. Each participant’s ID number and response is listed.
